# Comparison of live and fixed cell-based assay performance: implications for the diagnosis of MOGAD in a low-middle income country

**DOI:** 10.3389/fimmu.2023.1252650

**Published:** 2023-08-29

**Authors:** Lekha Pandit, Anitha D’Cunha, Chaithra Malli, Akshatha Sudhir

**Affiliations:** Center for Advanced Neurological Research, Nitte University, Mangalore, India

**Keywords:** MOGAD, international diagnostic criteria, live cell-based assay, fixed cellbased assay, assay performance

## Abstract

**Background:**

Though considered optimal, live cell-based assay (LCBA) is often unavailable for the diagnosis of myelin oligodendrocyte glycoprotein antibody-associated disorders (MOGAD) in resource-poor regions. This study was undertaken to determine the agreement between LCBA and the widely available fixed cell-based assay (FCBA), for recommending testing guidelines within our region.

**Method:**

All consecutive patients in our registry with a MOGAD phenotype were tested. The results from a commercially available FCBA (Euroimmun, Germany) were compared with a validated “in-house” LCBA. Clinical and MRI data were available for correlation.

**Results:**

Among the 257 patient samples tested, 118 (45.9%) were positive by FCBA titre ≥1: 10 and or LCBA titres ≥1: 160 titre and 139 samples were negative. There was robust agreement between the two assays (agreement 98.8%, Cohen’s kappa 0.98 [95% CI- 0.95-1.00], Spearman correlation 0.97 (p < 0.0001). Among five discordant samples, four had clinical and or MRI data which supported an alternate diagnosis. There was a modest correlation between assay titres, particularly for samples with titres ≥ 1:100 in FCBA (Spearman’s Rho 0.26, p 0.005). Thirty samples were positive by FCBA at < 1:100 titre and included 1:80 (20),1:40(7) and 1:10 (3) titres. Among them, 80% had clear positive titres when tested by LCBA.

**Conclusion:**

The FCBA tested with serum dilutions of 1:10 was highly predictive of MOGAD in our study and compared well with our “in-house” LCBA. The current recommendations for testing at higher dilutions need to be re-examined in light of our findings. The results of our study should ideally be replicated in a larger dataset but at the same time provide some guidance for the accurate diagnosis of MOGAD in resource-poor settings.

## Introduction

Myelin oligodendrocyte glycoprotein antibody-associated disorders (MOGAD) are a distinct group of idiopathic inflammatory central nervous system disorders (CNS). Clinically, there is significant overlap in presentation with other autoimmune CNS disorders such as multiple sclerosis (MS) and neuromyelitis optica spectrum disorder (NMOSD). However, there are striking differences in clinical course and magnetic resonance imaging (MRI) features (1–4). In 2007, an antibody targeting MOG was first detected using a sensitive radiolabelled assay that identified a set of patients with acute disseminated encephalitis (ADEM) (5). In recent years, it has been replaced by well-designed new-generation cell-based assays (CBA) that expanded the clinical spectrum of these disorders. Assays were found to be more sensitive and specific when the full conformational form of MOG protein was used as substrate and testing for IgG isotype of MOG protein (using IgG Fc or IgG1-specific secondary antibody) was done (6–12).

Comparative studies that evaluated assay performance for MOG IgG detection suggested that live CBA may be superior to fixed CBA (10, 12). In these studies, both live and fixed CBAs done at multiple centres were compared, using a limited number of samples from patients with either a pre-defined MOG IgG status or a known MOGAD phenotype (10, 12). A recent study that compared assay performances in 322 patient samples found an excellent agreement between LCBA and FCBA for the diagnosis of MOGAD (13). The newly developed international MOGAD panel proposed diagnostic criteria that not only emphasised the clinical phenotypes associated with MOGAD but also laid down criteria for interpretation of CBA (both LCBA and FCBA) for definitive diagnosis (14). Supportive criteria that included clinical and or MRI features were mandatory when assay results were inconclusive or assay titres were unknown.

In India where this study was done, the gross national income per capita is only 2380 $ (www.worldbank.org,2022). The majority of the population are uninsured and the cost of medical care is met by “out-of-pocket spending”. The technical skills and infrastructure needed to develop and maintain an LCBA are not commonly available in resource-poor regions. Commercially developed FCBA (Euroimmun, Lubeck, Germany) is available in diagnostic laboratories in larger metropolises, where testing is done in standard 1: 10 (serum) dilutions as per the manufacturer’s instructions. The testing of positive samples at higher dilutions, as recommended by the international criteria, will incur additional costs and potentially discourage diagnostic testing for MOGAD. In this context, the implications of diagnosing MOGAD using FCBA need to be determined.

## Method

We enrolled 257 consecutive patients who presented with a MOGAD phenotype in the Mangalore demyelinating disease registry [MANDDIR] (15) (**Table 1**). Demographic, clinical and magnetic resonance imaging (MRI) data were available. Patients had previously tested negative for AQP4-IgG, tested by LCBA assay in our lab (16).

**Table 1:** Clinical phenotype of patients (n= 257).

	MOG -IgG positive (n/%)	MOG-IgG negative (n/%)
PATIENT NUMBER	118 (45.9%)	139 (54.1%)
AGE (MEAN ± SD)	32.50 ± 14.67	36.64 ± 14.13
PEDIATRIC PATIENTS	19 (16%)	11 (7.9%)
DISEASE PHENOTYPE		
ON	60 (50.8%)	62 (44.6%)
UNILATERAL ON	18	47
BILATERAL ON	23	7
RECURRENT ON	19	8
TM	36 (30.5%)	41 (29.5%)
RECURRENT TM	2 (1.7%)	7 (5%)
BRAINSTEM	4 (3.5%)	1 (0.7%)
SYMPTOMATIC BRAIN LESION	0	2 (1.4%)
AQP4-IG NEGATIVE NMOSD	11 (9.3%)	26 (18.7%)
CORTICAL ENCEPHALITIS	1 (0.8%)	0
ADEM	4 (3.4%)	0

### Testing by FCBA

Serum samples were originally collected within 4 weeks of a recent/first attack, aliquoted and stored at -80°C. Initial testing was done with the commercially available FCBA as per the manufacturer’s instructions, within a median interval of 18 days from the date of collection. For the purpose of this study, clinical data was anonymized and all samples that tested positive in standard 1:10 dilution were further tested at 2fold dilutions (1:20,1:40 etc) and additionally at 10fold dilutions (1:100, 1:10,000&1:100000). A titer of ≥ 1:100 was determined to be clear positive and < 1:100 as low positive according to the current recommendations (14).

### Testing by LCBA

#### Development and validation of “in-house” LCBA for MOGAD

Full-length MOG was co-expressed with a fluorescent protein (MOG-EmGFP) on Chinese hamster ovary cells (CHO K1) that were transiently transfected (Lipofectamine™3000, Thermo Fisher Scientific, Germany) using a custom-made recombinant expression vector (EF1alpha-Human-FL-MOG-IRES-EmGFP-v1[Genscript]). Samples were added in 2 fold dilutions, starting at 1:10 dilution, incubated at room temperature for 2 hours, followed by washing and the addition of secondary antibody (1:1000 diluted Alexa Fluor^®^594-conjugated anti-human IgG Fcγ Fragment specific [Jackson Immunoresearch]). After a 45-minute incubation period and wash, DAPI mountant was added and the wells were observed under a fluorescent microscope (Nikon, Japan). The endpoint titer was determined as the highest dilution that demonstrated positive fluorescence. Clear positive titer was defined as two doubling dilutions above (≥1:640) and low positive titer as 1 doubling dilution (160 – 320) above assay cut-off (14, 17). The assay was validated (**Supplementary Figures 1, 2**) in 199 anonymized patient samples including those with a MOGAD phenotype (158), multiple sclerosis (20), and other neurological disorders (10) and healthy donors (11). Our assay showed high agreement when compared with the results of these samples tested earlier by an LCBA in Japan (agreement 99.5%, Cohen’s kappa 0.99 [95% CI 0.96- 1.01], Spearman correlation coefficient 0.98, p < 0.0001) (7, 18, 19).

#### Testing samples by LCBA

All patient samples with a MOGAD phenotype were anonymized, and aliquoted samples were thawed and tested as outlined earlier. The test results were independently read and scored by authors (AD and CM) well versed in the setting up and interpretation of a CBA (20). Differences in scoring were observed in five samples (2%) and resolved through consensus. We repeated the assays for eight samples that should show a marked discrepancy (low positive result in one assay with clear positive results and high titers in the other) in the assay titers. After completion, we deanonymized the patients’ identities and accessed their clinical and MRI data for further analysis. 

#### Statistics

Statistical analysis was performed using SPSS statistics (IBM Corp, Armonk, NY, USA). We used Cohen’s kappa coefficient to evaluate the concordance between the FCBA and LCBA. Correlations were assessed by Spearman’s Rho (p). A p-value of < 0.05 was considered statistically significant.

#### Protocol approvals and patient consent

This study was approved by the institutional ethics committee and signed informed consent was obtained from patients as per protocols of the Mangalore Demyelinating Disease Registry (MANDDIR).

## Results

**Figure 1:**
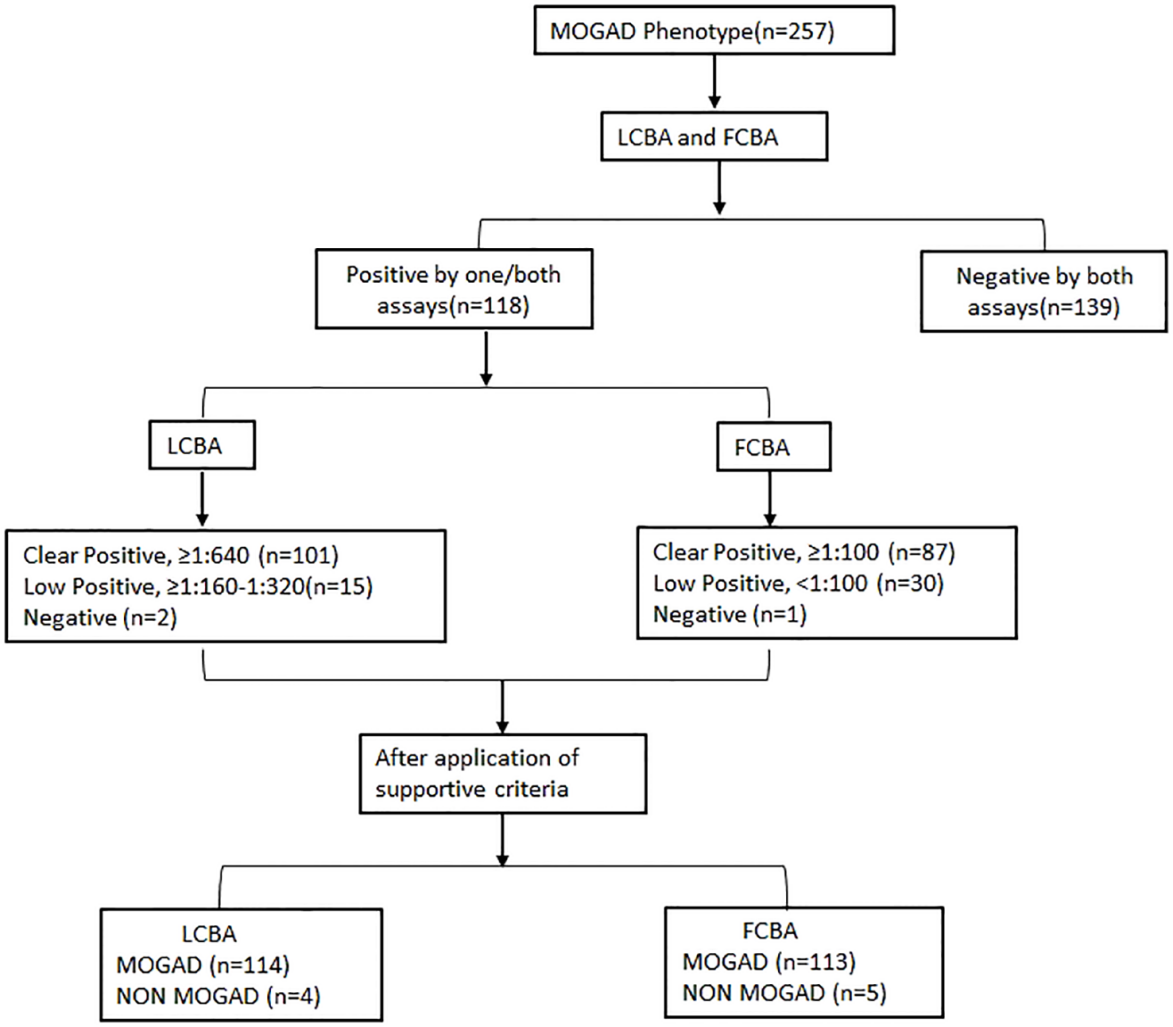
Flowchart showing results of both assays and the final diagnosis. MOGAD, Myelin oligodendrocyte glycoprotein antibody-associated disease; LCBA, Live cell-based assay; FCBA, Fixed cell-based assay.

An overview of assay results and the final diagnosis after adjusting against clinical/supportive criteria is outlined in **Figure 1**.

### Assay titres – Clear positive, negative and low titres for both assays

**Figure 2:**
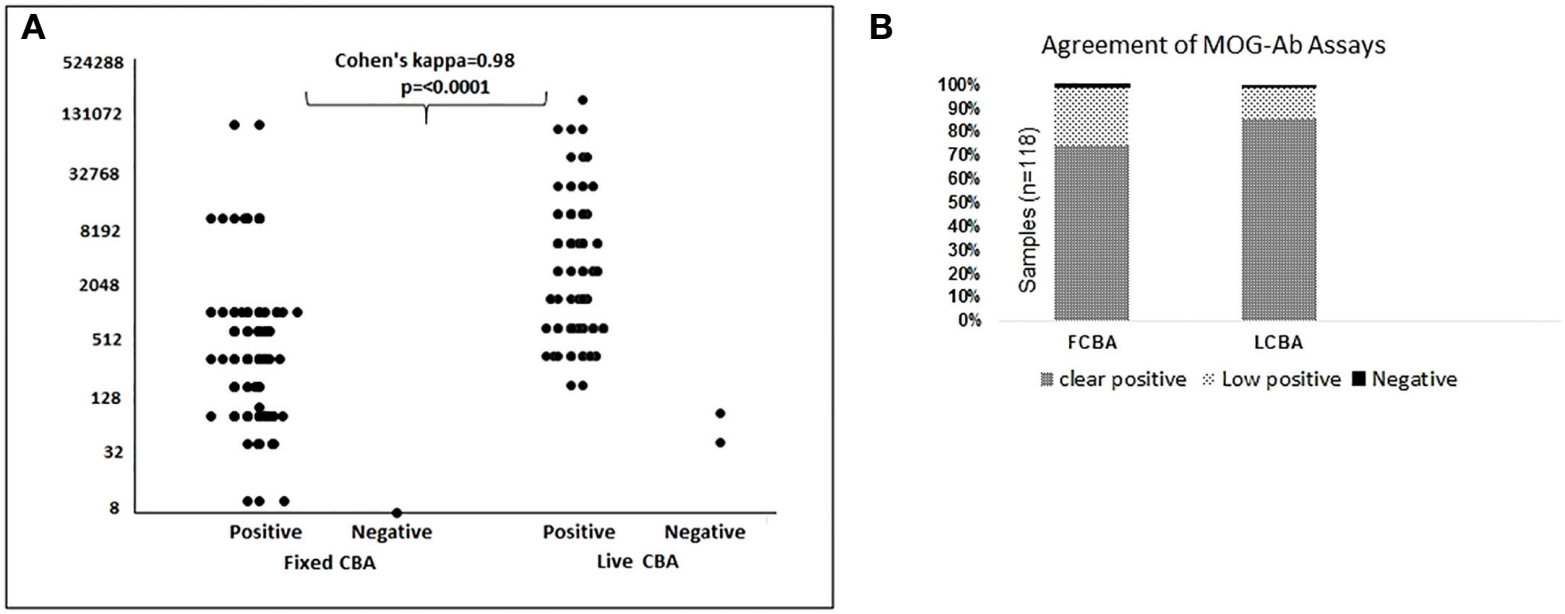
Agreement between fixed cell-based assay (FCBA) and live cell-based assay (LCBA) for MOGAD. **(A)** Agreement between FCBA (≥:10) and LCBA (≥1:160). **(B)** Percentage of samples that tested clear positive, low positive or negative by both assays.

Among the 257 patient samples tested, 118 (45.9%) tested positive by FCBA (≥1: 10) and/or LCBA (≥ 160) and 139 samples were negative. One sample tested positive by LCBA alone and 2 samples by FCBA. Overall agreement between the assays was 98.8%, Cohen’s kappa 0.98 [95% CI- 0.95-1.00], and Spearman’s correlation 0.97 (p < 0.0001) (**Figure 2**). Clear positive results (≥ 640 on LCBA/≥ 1:100 on FCBA) were obtained in 87 samples and 76 were concordant for both assays (**Supplementary Table 1**). Agreement between the assays was 85.9%, Cohen’s kappa 0.69 [95% CI 0.61- 0.78], and Spearman’s correlation 0.70 (p < 0. 0001).

### Live cell-based assay results

In total, 101 samples tested clear positive at titres ≥ 1:640. Furthermore, 15 samples tested low positive by LCBA (1:160 titre -7, 1: 320 titre - 8) and 2 samples were negative. The positive predictive value (PPV), calculated as true positive/positive samples (114/116) was 98.3% overall. At titres ≥ 1:640 (100/101) PPV was 99.01% and at titres ≥ 1:1280 (70/70) it was 100%.

### Fixed cell-based assay results

In total, 87 samples were clear positive, 30 were low positive and 1 was negative. Among the low positive results, 20 tested positive in 1:80 dilution, 7 in 1:40, and 3 in 1:10 dilution. The PPV for all tested samples was 96.6% (113/117), at 1: 100 dilution 97.7% (85/87) and at 1:320 it was 100% (77/77).

**Figure 3:**
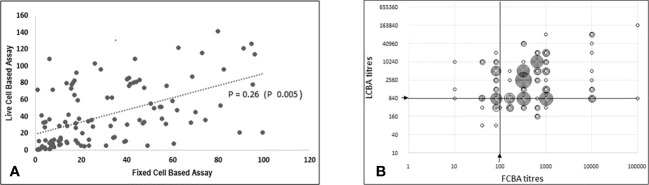
Correlation between titres from fixed cell-based assay and live cell-based assay results. **(A)** Scatter plot (using Excel CORREL function) with ranked FCBA titres on X axis and corresponding titres from LCBA on Y axis; Spearman's Rho-0.26(p 0.005). **(B)** Scatter plot (raw data) showing FCBA titres and corresponding results from LCBA plotted on X and Y axis respectively.

There was a modest correlation (Spearman’s Rho - 0.26, p 0.005) in antibody titres between both assays (**Figure 3**). Samples with low assay titres were particularly discordant. A repeat testing of some of these discordant samples did not change the final results. Among the low positive results from the FCBA, 24/30 (80%) samples had corresponding titres in the clear positive range in the LCBA. Similarly, 11/15 samples (73.3%) that had low positive results from the LCBA had clear positive results in FCBA testing (**Supplementary Tables 1, 2**). All 15 patients with low positive results from the LCBA and 28 patients positive by FCBA satisfied the supportive clinical and or MRI criteria. As outlined in the flow chart (**Figure 1**), four patients with a clinical phenotype for MOGAD (**Supplementary Table 3**) and a positive MOG-IgG test were excluded from the final list due to other etiologies. One patient with a MOGAD phenotype (recurrent optic neuritis and MRI documenting long segment optic neuritis) tested negative on the FCBA (the final tally of negative cases was four for the LCBA and five for the FCBA).

## Discussion

The diagnosis of MOGAD poses many clinical challenges because of varied presentations that overlap with those of MS and NMOSD. Additionally, there are limitations in accessing and interpreting diagnostic tests, particularly in resource-constrained regions. The newly developed international MOGAD panel criteria draw attention to the common clinical phenotypes, the accompanying imaging characteristics of this disease and the requirement for cell-based assays for definitive diagnosis (14). Some of the earlier comparative studies that evaluated the performance of different assays found that FCBAs showed lower agreement and PPV when compared with LCBAs (10, 12). It has been suggested the process of cell fixation could potentially damage the integrity of transfected cells, generate cryptic epitopes, or cause the loss of conformational epitopes, all of which have the potential to interfere with assay results (12, 21). The infrastructure and technical expertise required to develop LCBAs are limited in our region and most patients referred to our registry have been tested by the widely available commercial FCBA kit. In this context, we set out to understand how the commercially available FCBA compared with our validated “in-house” assay in testing patients from our registry. Two-fold and 10-fold serum dilutions were used for testing FCBA in order to determine whether lower dilutions misdiagnosed MOGAD and also to understand the range and correlation between assay titres. We relied on clinical, MRI, and other relevant tests to finalize the diagnosis.

There was excellent agreement between the assays at low titres: FCBA (1:10) and our “in-house” LCBA (≥1:160) titres. The agreement was less remarkable when only clear positive samples were compared. The diagnosis could be resolved in all 30 patients with low titre results (<1:100) on the FCBA when supportive criteria were applied (**Supplementary Table 2**). In total, 28 patients (93%) were diagnosed to have MOGAD and the remaining 2 were assigned an alternate diagnosis. All patients with low titre results on the LCBA had their diagnosis similarly resolved with the help of supportive criteria. Fourteen patients were diagnosed with MOGAD while one had an alternate diagnosis. False positive results were predictably seen with low titre samples as with previous reports (22). In addition, we had two patients who had high titres for MOG-IgG in one or both assays that required the help of supportive criteria for establishing an alternative diagnosis. With the exception of one patient who was negative in the FCBA, the diagnosis remained unchanged for the remaining 117 patients irrespective of the assay type.

There was modest correlation in assay titres and this was more evident in samples that tested clear positive by FCBA. Among the latter, 87.3% (76/87) had clear positive titres on LCBA. Notably, 80% (24/30) of patients with low titre results in the FCBA also had high titres (≥ 1:640) in the LCBA. These findings have significant implications for the interpretation of FCBA results and question the necessity for further testing at higher dilutions. Patient selection is important in order to improve pre-test probability and eliminate false positive results (22, 23). There are several potential factors that could have interfered with assay results including the timing of blood sampling and testing in relation to a recent attack/relapse, and we made efforts to minimise the same. Only 46% of patients with a MOGAD clinical phenotype tested positive for MOG-IgG, leaving open the possibility of other novel autoantibodies being associated with double seronegative non-MS disorders.

In conclusion, the experience from our registry unequivocally demonstrated the efficiency of FCBA tests for MOG-IgG detection in 1:10 serum dilution and for MOGAD diagnosis. The majority of our patients with low MOG IgG titres on FCBA had high (clear positive) titres on corresponding LCBA results. This has been emphasised in a recent study that found a high level of agreement between assays, but while titres were comparable, they were not identical (13). Testing at higher serum dilutions, as recommended in the international criteria, did not contribute additionally to the diagnosis. Detailed attention to phenotype matching and the judicious use of supportive features that accompany MOGAD diagnostic criteria were equally important for adjudicating the results of MOG-IgG serology and in the diagnosis of MOGAD. Replication of our results in a larger study may help to bolster our testing recommendations for the diagnosis of MOGAD.

## Data availability statement

The original contributions presented in the study are included in the article/**Supplementary Material**. Further inquiries can be directed to the corresponding author.

## Ethics statement

The studies involving humans were approved by Central Ethics Committee,Nitte University. The studies were conducted in accordance with the local legislation and institutional requirements. The participants provided their written informed consent to participate in this study.

## Author contributions

LP developed the concept, study design, analysis,interpretation,drafting and revision of the work. AD’C contributed to study design, data acquisition, analysis,interpretation, manuscript drafting and revision. CM contributed to data aquisition, analysis, interpretation of results and manuscript revision. AS contributed to data collection, data analysis and interpretation. All authors contributed to the article and approved the submitted version.
