# Supplementary material for: Comparison of live and fixed cell-based assay performance: implications for the diagnosis of MOGAD in a low-middle income country
Source: Front Immunol. 2023 Aug 29;14:1252650. doi: 10.3389/fimmu.2023.1252650 (PMC10495565; doi:10.3389/fimmu.2023.1252650)
Supplement: Supplementary file 1 [file DataSheet_1.docx]

SUPPLEMENTARY MATERIAL

Table 1: Master table with all sample results from the FCBA, “in-house” assay, results of some samples tested in Japan^18^ , and corresponding clinical phenotypes

|  |  | FIXED CELL-BASED ASSAY | | | | | | | | | LIVE ASSAY | |
| --- | --- | --- | --- | --- | --- | --- | --- | --- | --- | --- | --- | --- |
| SI NO | PHENOTYPE | 1:100000 | 1:10000 | 1: 1000 | 1: 160 | 1:100 | 1:80 | 1:40 | 1:20 | 1:10 | “In-house”  ASSAY | JAPAN^18^ ASSAY |
| 1 | TM* | - | - | - | - | - | + | + | + | + | 1: 1 2 8 0 | + |
| 2 | BS* | - | - | - | - | - | + | + | + | + | 1: 6 4 0 | + |
| 3 | RTM | - | - | + | + | + | + | + | + | + | 1:20480 | + |
| 4 | TM | - | + | + | + | + | + | + | + | + | 1:20480 | + |
| 5 | TM | - | - | - | + | + | + | + | + | + | 1: 6 4 0 | + |
| 6 | RON | - | - | - | - | - | + | + | + | + | 1: 6 4 0 | + |
| 7 | RON | - | - | - | + | + | + | + | + | + | 1: 5 1 20 | + |
| 8 | TM | - | - | - | - | - | + | + | + | + | 1:10240 | + |
| 9 | TM* | - | - | - | - | - | - | + | + | + | 1:10240 | + |
| 10 | TM | - | - | + | + | + | + | + | + | + | 1 : 1 2 8 0 | + |
| 11 | TM | - | - | - | + | + | + | + | + | + | 1:40960 | + |
| 12 | TM | - | - | - | + | + | + | + | + | + | 1:2560 | + |
| 13 | ON BILATERAL | - | - | - | - | - | - | + | + | + | 1 : 6 4 0 | + |
| 14 | ON BILATERAL | - | - | - | + | + | + | + | + | + | 1:20480 | + |
| 15 | RON | - | - | - | + | + | + | + | + | + | 1:20480 | + |
| 16 | RON | + | + | + | + | + | + | + | + | + | >1:63840 | + |
| 17 | RON | - | + | + | + | + | + | + | + | + | 1 : 6 4 0 | + |
| 18 | ON BILATERAL | - | - | - | + | + | + | + | + | + | 1:5120 | + |
| 19 | RON | - | - | - | + | + | + | + | + | + | 1:2560 | + |
| 20 | TM | - | - | - | - | - | + | + | + | + | 1 : 3 2 0 | + |
| 21 | TM | - | - | - | + | + | + | + | + | + | 1:2560 | + |
| 22 | ON BILATERAL | - | - | + | + | + | + | + | + | + | 1:5120 | + |
| 23 | NMOSD | - | - | + | + | + | + | + | + | + | 1 : 1 2 8 0 | + |
| 24 | RTM | - | - | - | + | + | + | + | + | + | 1:2560 | + |
| 25 | TM | - | - | - | + | + | + | + | + | + | 1:10240 | + |
| 26 | TM | - | - | - | - | - | + | + | + | + | 1:5120 | + |
| 27 | TM | - | - | - | - | - | - | + | + | + | 1 : 6 4 0 | + |
| 28 | ON BILATERAL | - | - | - | + | + | + | + | + | + | 1 : 1 2 8 0 | + |
| 29 | RON | - | - | - | + | + | + | + | + | + | 1:10240 | + |
| 30 | NMOSD | - | - | - | + | + | + | + | + | + | 1:40960 | + |
| 31 | TM | - | - | + | + | + | + | + | + | + | 1 : 6 4 0 | + |
| 32 | TM | - | - | - | - | - | - | - | - | + | 1 : 1 2 8 0 | + |
| 33 | ON BILATERAL | - | - | - | - | - | + | + | + | + | 1:2560 | + |
| 34 | NMOSD | - | - | - | + | + | + | + | + | + | 1:5120 | + |
| 35 | ADEM | - | - | - | - | - | + | + | + | + | 1 : 6 4 0 | + |
| 36 | TM | - | - | - | - | - | + | + | + | + | 1:5120 | + |
| 37 | RON | - | - | - | + | + | + | + | + | + | 1:5120 | + |
| 38 | TM | - | - | - | - | - | - | + | + | + | 1 : 3 2 0 | ND |
| 39 | RON | - | - | + | + | + | + | + | + | + | 1:5120 | ND |
| 40 | ON BILATERAL | - | - | - | + | + | + | + | + | + | 1 : 6 4 0 | ND |
| 41 | ON BILATERAL | - | - | + | + | + | + | + | + | + | 1 : 6 4 0 | ND |
| 42 | ON BILATERAL | - | - | - | + | + | + | + | + | + | 1 : 6 4 0 | ND |
| 43 | ON | - | - | - | + | + | + | + | + | + | 1 : 3 2 0 | ND |
| 44 | TM | - | - | - | + | + | + | + | + | + | 1 : 6 4 0 | ND |
| 45 | RON | - | - | + | + | + | + | + | + | + | 1 : 6 4 0 | ND |
| 46 | ON BILATERAL | - | - | - | + | + | + | + | + | + | 1 : 6 4 0 | ND |
| 47 | TM | - | - | + | + | + | + | + | + | + | 1 : 6 4 0 | ND |
| 48 | NMOSD | - | - | - | - | - | + | + | + | + | 1 : 6 4 0 | ND |
| 49 | BS | - | - | - | - | - | + | + | + | + | 1:5120 | ND |
| 50 | NMOSD | - | - | - | + | + | + | + | + | + | 1:5120 | ND |
| 51 | ON BILATERAL | - | - | - | + | + | + | + | + | + | 1 : 6 4 0 | ND |
| 52 | TM | - | - | - | + | + | + | + | + | + | 1 : 3 2 0 | ND |
| 53 | RON | - | - | + | + | + | + | + | + | + | 1 : 6 4 0 | ND |
| 54 | TM | + | + | + | + | + | + | + | + | + | 1 : 6 4 0 | ND |
| 55 | RON | - | - | - | + | + | + | + | + | + | 1 : 1 6 0 | + |
| 56 | NMOSD | - | - | + | + | + | + | + | + | + | 1:5120 | ND |
| 57 | NMOSD | - | - | - | + | + | + | + | + | + | 1:2560 | ND |
| 58 | RON | - | - | - | + | + | + | + | + | + | 1 : 3 2 0 | ND |
| 59 | ON BILATERAL | - | - | - | + | + | + | + | + | + | 1 : 3 2 0 | ND |
| 60 | ON BILATERAL | - | - | - | + | + | + | + | + | + | 1 : 3 2 0 | ND |
| 61 | RON | - | - | - | - | - | + | + | + | + | 1:2560 | ND |
| 62 | NMOSD | - | - | - | + | + | + | + | + | + | 1:2560 | ND |
| 63 | ADEM | - | - | - | + | + | + | + | + | + | 1:5120 | ND |
| 64 | RON | - | - | + | + | + | + | + | + | + | 1 : 3 2 0 | ND |
| 65 | ON | - | - | - | + | + | + | + | + | + | 1 : 6 4 0 | ND |
| 66 | TM | - | - | - | - | - | + | + | + | + | 1 : 3 2 0 | ND |
| 67 | RON | - | - | - | + | + | + | + | + | + | 1:2560 | ND |
| 68 | ON | - | + | + | + | + | + | + | + | + | 1:2560 | ND |
| 69 | ON | - | + | + | + | + | + | + | + | + | 1:5120 | ND |
| 70 | ON BILATERAL | - | - | + | + | + | + | + | + | + | 1:10240 | ND |
| 71 | ON | - | - | + | + | + | + | + | + | + | 1:2560 | ND |
| 72 | ON | - | + | + | + | + | + | + | + | + | 1 : 1 2 8 0 | ND |
| 73 | ON BILATERAL | - | - | - | + | + | + | + | + | + | 1:2560 | ND |
| 74 | ON | - | - | - | - | - | + | + | + | + | 1 : 3 2 0 | ND |
| 75 | TM | - | - | - | + | + | + | + | + | + | 1:10240 | ND |
| 76 | CORTICAL ENCEPHALITIS | - | - | - | + | + | + | + | + | + | 1 : 1 6 0 | ND |
| 77 | TM | - | - | - | - | - | + | + | + | + | 1:20480 | ND |
| 78 | ON | - | + | + | + | + | + | + | + | + | 1:40960 | ND |
| 79 | ON | - | - | + | + | + | + | + | + | + | 1:20480 | ND |
| 80 | NMOSD | - | - | - | + | + | + | + | + | + | 1 : 6 4 0 | ND |
| 81 | ON BILATERAL | - | + | + | + | + | + | + | + | + | 1 : 6 4 0 | ND |
| 82 | ON BILATERAL | - | - | - | + | + | + | + | + | + | 1:10240 | ND |
| 83 | ON BILATERAL | - | - | + | + | + | + | + | + | + | 1:81920 | ND |
| 84 | ADEM | - | - | + | + | + | + | + | + | + | 1 : 6 4 0 | ND |
| 85 | ON | - | - | - | + | + | + | + | + | + | 1:10240 | ND |
| 86 | ON | - | - | - | + | + | + | + | + | + | 1:10240 | ND |
| 87 | TM | - | - | - | + | + | + | + | + | + | 1 : 6 4 0 | ND |
| 88 | ON BILATERAL* | - | - | - | - | - | + | + | + | + | 1 : 1 2 8 0 | ND |
| 89 | TM | - | - | - | - | - | + | + | + | + | 1:10240 | + |
| 90 | TM* | - | - | - | - | - | - | - | - | + | 1:10240 | ND |
| 91 | TM* | - | - | - | - | - | - | + | + | + | 1:10240 | ND |
| 92 | TM | - | - | - | + | + | + | + | + | + | 1:5120 | ND |
| 93 | ON BILATERAL | - | - | - | + | + | + | + | + | + | 1 : 3 2 0 | ND |
| 94 | RON | - | - | - | - | - | + | + | + | + | 1 : 6 4 0 | ND |
| 95 | TM | - | + | + | + | + | + | + | + | + | 1:81920 | ND |
| 96 | ON | - | - | - | + | + | + | + | + | + | 1:5120 | ND |
| 97 | ON | - | - | - | + | + | + | + | + | + | 1:2560 | ND |
| 98 | NMOSD | - | - | + | + | + | + | + | + | + | 1:20480 | ND |
| 99 | ON BILATERAL | - | - | - | + | + | + | + | + | + | 1:5120 | ND |
| 100 | TM | - | - | + | + | + | + | + | + | + | 1:10240 | ND |
| 101 | TM | - | - | - | + | + | + | + | + | + | 1:10240 | ND |
| 102 | ON | - | - | - | - | - | - | + | + | + | 1 : 6 4 0 | ND |
| 103 | ON BILATERAL | - | - | - | + | + | + | + | + | + | 1 : 1 2 8 0 | ND |
| 104 | ON BILATERAL | - | - | - | + | + | + | + | + | + | 1:2560 | ND |
| 105 | ON | - | - | - | - | - | + | + | + | + | 1:5120 | ND |
| 106 | RON | - | - | - | + | + | + | + | + | + | 1 : 6 4 0 | ND |
| 107 | TM | - | + | + | + | + | + | + | + | + | 1 : 6 4 0 | ND |
| 108 | RON | - | - | - | + | + | + | + | + | + | 1 : 3 2 0 | ND |
| 109 | ON* | - | - | - | - | - | - | - | - | - | 1 : 6 4 0 | ND |
| 110 | BS | - | - | - | + | + | + | + | + | + | 1 : 512 0 | ND |
| 111 | TM | - | + | + | + | + | + | + | + | + | 1:81920 | ND |
| 112 | TM | - | - | - | - | - | - | - | - | + | 1: 6 4 0 | + |
| 113 | NMOSD | - | - | - | - | + | + | + | + | + | 1 : 1 2 8 0 | + |
| 114 | ADEM | - | - | - | - | + | + | + | + | + | 1 : 1 2 8 0 | + |
| 115 | ON | - | - | - | - | + | + | + | + | + | 1: 6 4 0 | ND |
| 116 | BS | - | - | - | - | + | + | + | + | + | 1 : 3 2 0 | ND |
| 117 | ON | - | - | - | - | - | + | + | + | + | 1 : 80 | ND |
| 118 | TM | - | - | - | - | - | - | + | + | + | 1 : 80 | ND |

Abbreviations : TM- transverse myelitis, ON- optic neuritis, BS-brainstem , ADEM- acute disseminated encephalomyelitis,NMOSD- neuromyelitis optica spectrum disorder, ND-not done

Samples(n=7) that were rechecked by both assays are marked by an asterix against the clinical phenotype

Table 2. Low positive results on either assay – clinical phenotype and supportive features

| No: | CLINICAL PHENOTYPE | FIXED CELL-BASED ASSAY | | | | | | | | | | | “In-house”  LCBA | SUPPORTIVE CRITERIA Clinical/MRI findings |
| --- | --- | --- | --- | --- | --- | --- | --- | --- | --- | --- | --- | --- | --- | --- |
|  |  | 1:1000000 | 1:10000 | 1: 1000 | 1 : 640 | 1 : 320 | 1:160 | 1: 100 | 1:80 | 1 :40 | 1 :20 | 1 :10 |  |  |
| **1** | **TM** | - | - | - | - | - | - | - | + | + | + | + | 1 : 3 2 0 | LETM |
| **2** | **TM** | - | - | - | - | - | - | - | - | + | + | + | 1 : 3 2 0 | LETM |
| 3 | TM | - | - | - | + | + | + | + | + | + | + | + | 1 : 3 2 0 | LETM & nonspecific subcortical brain lesions |
| 4 | ON | - | - | - | - | - | + | + | + | + | + | + | 1 : 3 2 0 | Optic disc oedema, Long segment optic neuritis |
| 5 | ON BILATERAL | - | - | - | - | - | + | + | + | + | + | + | 1 : 3 2 0 | Bilateral long segment optic neuritis |
| 6 | ON BILATERAL | - | - | - | - | - | + | + | + | + | + | + | 1 : 3 2 0 | Bilateral long segment optic neuritis and optic sheath and orbital fat enhancement |
| 7 | ON | - | - | + | + | + | + | + | + | + | + | + | 1 : 3 2 0 | Optic disc oedema and long segment optic neuritis |
| **8** | **TM** | - | - | - | - | - | - | - | + | + | + | + | 1 : 3 2 0 | LETM and short segment myelitis |
| **9** | **ON** | - | - | - | - | - | - | - | + | + | + | + | 1 : 3 2 0 | Unilateral long segment optic neuritis sparing optic chiasm |
| 10 | CE | - | - | - | + | + | + | + | + | + | + | + | 1 : 1 6 0 | Bilateral frontal,temporal and parietal cortical lesions, subcortical lesions |
| 11 | ON BILATERAL | - | - | - | - | + | + | + | + | + | + | + | 1 : 3 2 0 | Long segment optic neuritis sparing optic chiasm |
| 12 | ON | - | - | - | + | + | + | + | + | + | + | + | 1 : 3 2 0 | Long segment optic neuritis with enhancement of optic nerve sheath |
| 13 | ON | - | - | - | - | + | + | + | + | + | + | + | 1 : 3 2 0 | Optic disc odema,long segment optic neuritis |
| 14 | ON | - | - | - | + | + | + | + | + | + | + | + | 1 : 1 6 0 | Optic disc odema,long segment optic neuritis |
| 15 | BS | - | - | - | - | + | + | + | + | + | + | + | 1 : 320 | Ill defined brainstem and bilateral middle cerebellar peduncle lesions |
| 16 | TM | - | - | - | - | - | - | - | + | + | + | + | 1 : 1 2 8 0 | LETM |
| 17 | BS | - | - | - | - | - | - | - | + | + | + | + | 1 : 6 4 0 | Ill defined brainstem lesions particularly around 4^th^ ventricle, subcortical lesions |
| 18 | ON | - | - | - | - | - | - | - | + | + | + | + | 1 : 6 4 0 | Optic disc oedema, long segment optic neuritis |
| 19 | TM | - | - | - | - | - | - | - | + | + | + | + | 1:10240 | LETM |
| 20 | TM | - | - | - | - | - | - | - | - | + | + | + | 1:10240 | LETM, ill defined subcortical lesions |
| 21 | ON BILATERAL | - | - | - | - | - | - | - | - | + | + | + | 1 : 6 4 0 | Bilateral long segment optic neuritis and optic sheath enhancement |
| 22 | TM | - | - | - | - | - | - | - | + | + | + | + | 1 : 51 2 0 | LETM ,conus myelitis |
| 23 | TM | - | - | - | - | - | - | - | - | + | + | + | 1 : 6 4 0 | LETM |
| 24 | TM | - | - | - | - | - | - | - | - | - | - | + | 1 : 1 2 8 0 | LETM |
| 25 | ON BILATERAL | - | - | - | - | - | - | - | + | + | + | + | 1 : 2560 | Optic disc oedema, bilateral optic neuritis |
| 26 | TM | - | - | - | - | - | - | - | + | + | + | + | 1 : 6 4 0 | LETM ,conus myelitis |
| 27 | BS | - | - | - | - | - | - | - | + | + | + | + | 1: 5120 | Brainstem lesions including floor of 4^th^ ventricle and middle cerebral peduncles. |
| 28 | ON | - | - | - | - | - | - | - | + | + | + | + | 1: 5120 | Long segment Optic nerve , punctate subcortical brain lesions |
| 29 | TM | - | - | - | - | - | - | - | + | + | + | + | 1 : 20480 | LETM |
| 30 | ON BILATERAL | - | - | - | - | - | - | - | + | + | + | + | 1 : 1 2 8 0 | Bilateral long segment optic neuritis |
| 31 | TM | - | - | - | - | - | - | - | + | + | + | + | 1 : 1 0240 | LETM and conus myelitis |
| 32 | TM | - | - | - | - | - | - | - | - | - | - | + | 1 : 1 0240 | LETM |
| 33 | TM | - | - | - | - | - | - | - | - | + | + | + | 1 : 1 0240 | LETM |
| 34 | ON | - | - | - | - | - | - | - | + | + | + | + | 1 : 6 4 0 | Optic disc oedema, long segment optic neuritis sparing chiasm, ill defined subcortical lesions |
| 35 | ON | - | - | - | - | - | - | - | - | + | + | + | 1 : 6 4 0 | Long segment optic neuritis, ill defined subcortical lesions, asymptomatic conus myelitis |
| 36 | ON | - | - | - | - | - | - | - | + | + | + | + | 1: 5120 | Long segment optic neuritis |
| 37 | ON | - | - | - | - | - | - | - | - | - | - | + | 1: 6 4 0 | Long segment optic neuritis, asymptomatic short segment myelitis |
| 38 | ADEM | - | - | - | - | - | - | - | + | + | + | + | 1 : 6 4 0 | Bilateral subcortical,thalamic and deep grey matter invovement |
| 39 | TM | - | - | - | - | - | - | - | + | + | + | + | 1: 5120 | LETM |
| 40 | ON | - | - | - | - | - | - | - | - | + | + | + | Negative | Features typical of MS |
| 41 | ON | - | - | - | - | - | - | - | - | - | - | + | Negative | Features typical of MS |

Abbreviations : TM- transverse myelitis, ON- optic neuritis, ADEM- acute disseminated encephalomyelitis, LETM- longitudinally extensive transverse myelitis, MS-multiple sclerosis

Bold serial numbers indicate patient samples which were “low positive”( <1:100 for FCBA and <640 for LCBA) for both assays.

Table 3:

Patients with other diseases with positive MOG-IgG serology

| No: | Age | Gender | phenotype | Supportive MRI | LCBA | FCBA |
| --- | --- | --- | --- | --- | --- | --- |
| 1 | 55 | F | Brainstem attack | MRI supportive of CVA | 1 : 320 | 1: 100 |
| 2 | 45 | F | Unilateral ON | MRI and CSF supportive of MS | negative | 1: 40 |
| 3 | 32 | F | Unilateral ON | MRI and CSF supportive of MS | negative | 1:20 |
| 4 | 33 | F | Unilateral ON | MRI and MR angiogram showed multiple hematomas and vasculitis respectively | 1: 640 | 1: 160 |
