# Supplementary figures and images for: Comparison of live and fixed cell-based assay performance: implications for the diagnosis of MOGAD in a low-middle income country
Source: Front Immunol. 2023 Aug 29;14:1252650. doi: 10.3389/fimmu.2023.1252650 (PMC10495565; doi:10.3389/fimmu.2023.1252650)

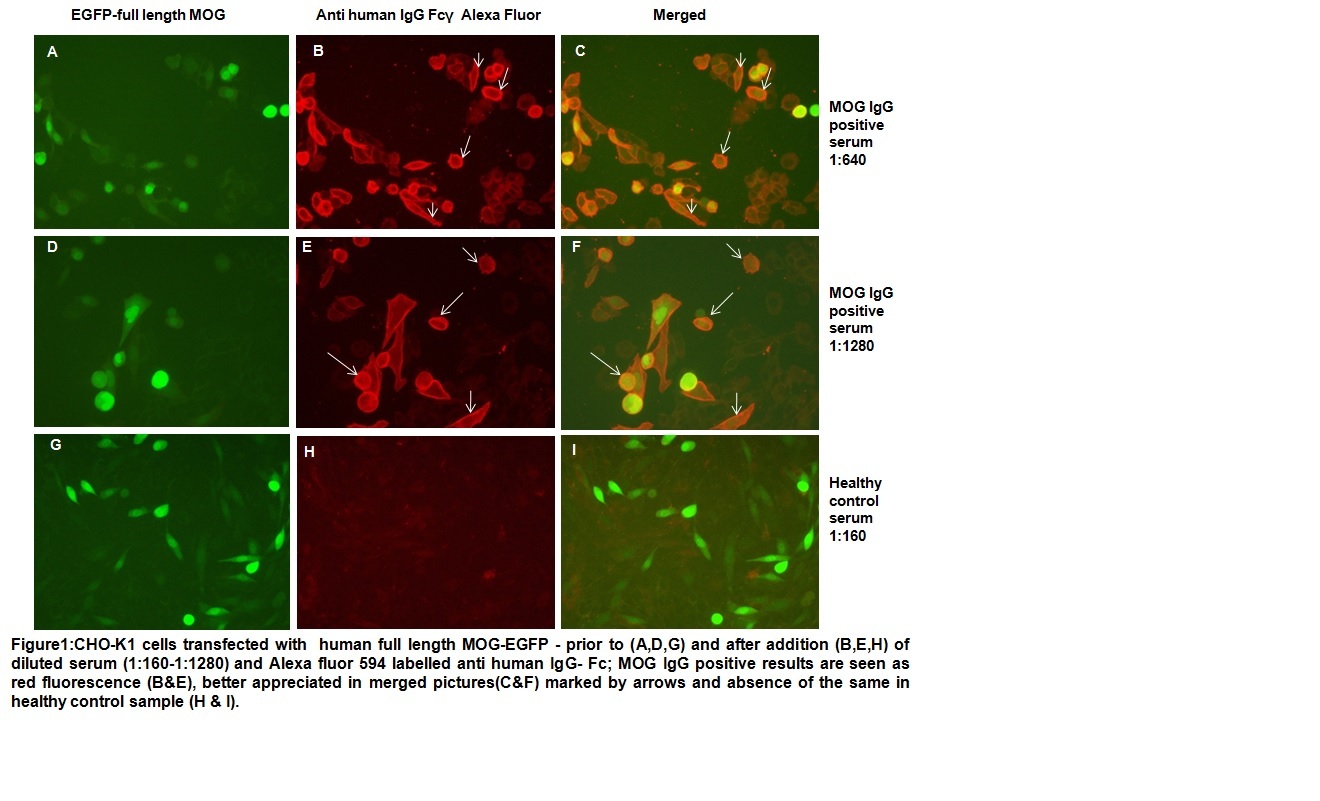

Supplement: Supplementary file 2 [file Image_1.jpeg]

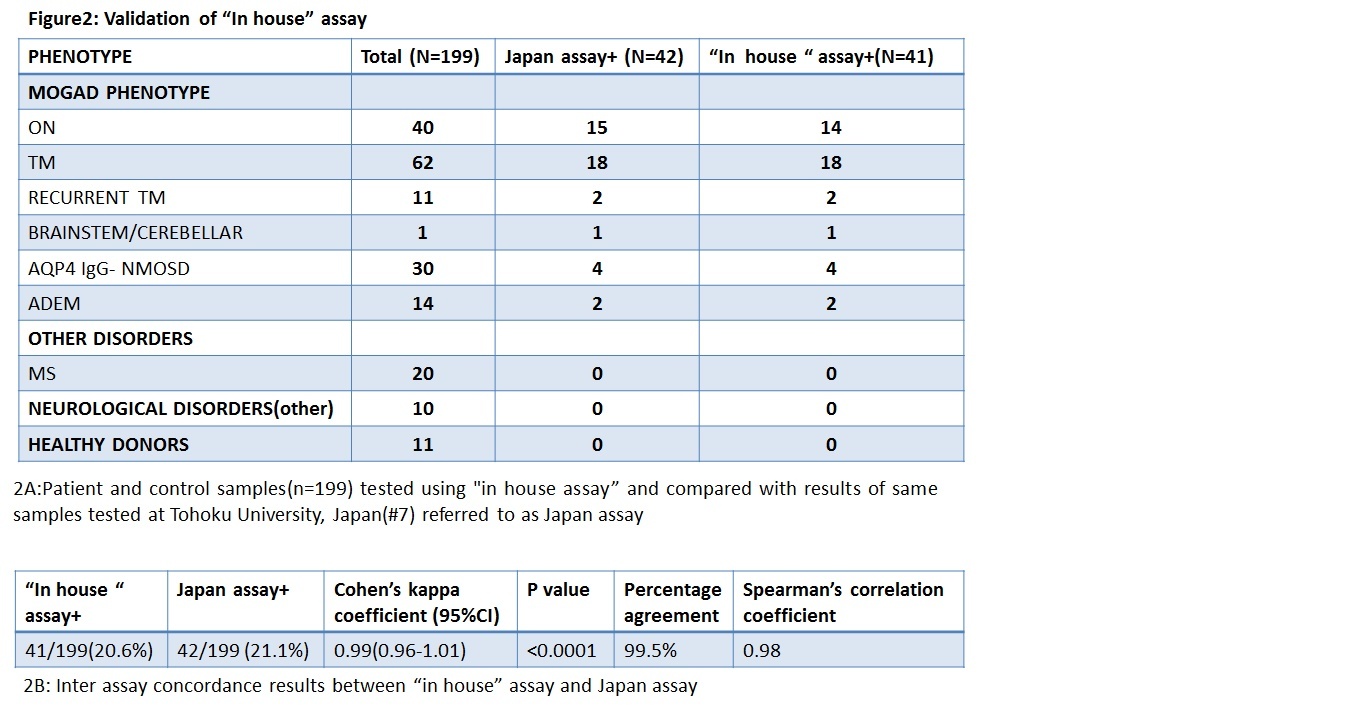

Supplement: Supplementary file 3 [file Image_2.jpeg]
